# Differential connectivity of the posterior piriform cortex in Parkinson’s disease and postviral olfactory dysfunction: an fMRI study

**DOI:** 10.1038/s41598-024-56996-1

**Published:** 2024-03-15

**Authors:** Charalampos Georgiopoulos, Martha Antonia Buechner, Bjoern Falkenburger, Maria Engström, Thomas Hummel, Antje Haehner

**Affiliations:** 1 Diagnostic Radiology, Department of Clinical Sciences, Medical Faculty, Lund University, Lund, Sweden; 2 Smell and Taste Clinic, Department of Otorhinolaryngology, TU Dresden, Dresden, Germany; 3 Department of Neurology, TU Dresden, Dresden, Germany; 4 Department of Health, Medicine, and Caring Sciences, Linköping University, Linköping, Sweden; 5 Center for Medical Image Science and Visualization (CMIV), Linköping University, Linköping, Sweden; 6 Department of Radiology, Section of Neuroradiology and Odontology, Skånes Universitetssjukhus, Entrégatan 7, 221 85 Lund, Sweden

**Keywords:** Smell, Olfaction, fMRI, Parkinson, COVID-19, Hyposmia, Parkinson's disease, Olfactory cortex

## Abstract

Olfactory dysfunction is a common feature of both postviral upper respiratory tract infections (PV) and idiopathic Parkinson’s disease (PD). Our aim was to investigate potential differences in the connectivity of the posterior piriform cortex, a major component of the olfactory cortex, between PV and PD patients. Fifteen healthy controls (median age 66 years, 9 men), 15 PV (median age 63 years, 7 men) and 14 PD patients (median age 70 years, 9 men) were examined with task-based olfactory fMRI, including two odors: peach and fish. fMRI data were analyzed with the co-activation pattern (CAP) toolbox, which allows a dynamic temporal assessment of posterior piriform cortex (PPC) connectivity. CAP analysis revealed 2 distinct brain networks interacting with the PPC. The first network included regions related to emotion recognition and attention, such as the anterior cingulate and the middle frontal gyri. The occurrences of this network were significantly fewer in PD patients compared to healthy controls (*p* = 0.023), with no significant differences among PV patients and the other groups. The second network revealed a dissociation between the olfactory cortex (piriform and entorhinal cortices), the anterior cingulate gyrus and the middle frontal gyri. This second network was significantly more active during the latter part of the stimulation, across all groups, possibly due to habituation. Our study shows how the PPC interacts with areas that regulate higher order processing and how this network is substantially affected in PD. Our findings also suggest that olfactory habituation is independent of disease.

## Introduction

Olfactory dysfunction is a hallmark of neurodegeneration, as it is associated with both Alzheimer’s and Parkinson’s disease (PD)^1^. In PD, olfactory dysfunction affects more than 90% of patients, often precedes motor symptoms and is independent of age, gender, treatment, duration and state of the disease^2^. However, olfactory dysfunction also manifests in patients with postviral upper respiratory tract infection (PV patients), as they experience sudden onset of anosmia or hyposmia subsequent to an upper respiratory tract infection^3^. As many as one third of PV patients experience an improvement in olfactory function within 12 months, however recovery is often incomplete^4^. The spotlight on postviral olfactory dysfunction has recently intensified, largely due to its association with the COVID-19 pandemic^5^, with some scientists discussing potential links between COVID-19 and PD^6^.

The impact that various disorders have on brain function can be investigated with functional magnetic resonance imaging (fMRI). By using the Blood Oxygen Level Dependent (BOLD) contrast to make inferences about neuronal activity, fMRI has been extensively employed for investigating the neural underpinnings associated with various brain functions, including olfaction. Previous studies have demonstrated impaired activation of olfactory-related brain regions in PD patients^7,8^. In the context of PV olfactory dysfunction, the application of fMRI has shown no significant differences in terms of olfactory brain activity between COVID-19 and other PV patients^9^.

Much like the diverse range of fMRI applications across various brain functions, there is a multitude of distinct analytical approaches. Traditionally, many studies have employed a general linear approach to map BOLD changes in response to specific stimulation paradigms^10–15^. Later, independent component analysis emerged as a model-free method for decomposing signal variance into different activation and artefactual components^16^. More recently, the focus has shifted towards dynamic analytical approaches, focusing on how brain regions change their interactions during an fMRI session^17^. One such approach involves selecting key time points that shape whole-brain correlations, reducing computational complexity, and yielding co-activation patterns (CAPs)^18^. CAP analysis is a frame-wise analytical tool that has already been employed in various cohorts, spanning conditions like bipolar disorder and autism^19,20^. Considering the dynamic nature of brain connectivity, where brain regions continuously reorganize their interactions over time, the CAP methodology provides a dynamic and detailed view of how specific brain regions interact with the rest of the brain during an fMRI session. By focusing on a select number of salient time points that hold significant information about brain connectivity, CAP reduces the need for extensive comparisons, thereby enhancing the statistical power of the analysis. This feature is especially beneficial in studies involving small sample sizes.

This study employed CAP analysis to investigate the dynamics of posterior piriform cortex connectivity across three diagnostic groups: healthy controls, PD patients, and PV patients with persistent olfactory dysfunction. The selection of this brain region as seed was motivated by its status as the largest structure within the primary olfactory cortex, located in the typically artifact-free temporal lobe^21^. Our hypothesis centered on the anticipation of identifying at least one network in which PD patients would exhibit significantly reduced activation, due to the longer-term impact on the posterior piriform cortex in PD compared to PV patients.

## Methods

### Participants

We recruited 15 healthy controls, 16 PD patients and 15 PV patients. Among PV patients, 13 had olfactory loss due to COVID-19. Exclusion criteria included claustrophobia or magnetic/electromagnetic implants (such as pacemakers). Written informed consent was obtained from all participants. The study was conducted in accordance with the 1964 Helsinki declaration and its later amendments and was approved by the local Ethical Review Authority (registration number EK 262082010).

Prior to MRI examination, all participants underwent olfactory assessment with the Sniffin’ sticks battery (Burghart Messtechnik GmbH, Holm, Germany), across three olfactory domains: threshold (T), discrimination (D) and identification (I), which combined yielded the global Threshold, Discrimination and Identification (TDI) score. Additionally, all participants were examined with the Montreal Cognitive Assessment (MoCA) test. PD and PV patients were also asked to subjectively rate how affected they felt from their impaired olfaction on a visual analogue scale (VAS) with its extreme left of the scale defined as "not at all" (0 units) and the extreme right of the scale defined as severely impaired olfaction (10 units; hereinafter termed as “subjective hyposmia”). PD patients were also clinically assessed with the Unified Parkinson’s Disease Rating Scale (UPDRS) and the Hoehn & Yahr scale during the ON state of the disease.

MRI examination included Susceptibility Weighted Imaging (SWI) of the nigrosome-1 (please see below). One PD patient was excluded as he demonstrated normal nigrosome-1 bilaterally and a lacunar infarct in the left caudate nucleus. Another PD patient was excluded due to severe motion artefacts during the fMRI session (> 3 mm). The remaining 14 PD patients, together with all 15 healthy controls and 15 PV patients were included in data analysis. Demographics are summarized in Table 1.

**Table 1:** Demographics (presented in the form of median value and interquartile range).

	Healthy controls (n = 15)	Parkinson’s disease (n = 14)	Postviral hyposmia (n = 15)
Age (years)	66 (64–77)	70 (63–73)	63 (60–70)
Sex	9 men	9 men	7 men
Threshold (T) score	6.5 (5–8.25)	1.63 (1.19–3.13)	3 (1.25–3.5)
Discrimination (D) score	13 (12–14)	8 (7–8.25)	10 (8–11)
Identification (I) score	13 (12–14)	6 (3.75–8)	9 (6–10)
Combined TDI score	31.25 (30.5–34.25)	16.25 (13.38–19.38)	21.75 (16.5–25.5)
Duration of olfactory dysfunction (months)	n.a	132 (24–192)	15 (9–16)
Subjective hyposmia (0–10)	n.a	2.9 (0.7–4)	7.3 (4–7.7)
MoCA	27 (26–29)	26 (21.75–28.25)	27 (26–28)
UPDRS	–	23 (17.5–36.5)	–
Hoehn and Yahr	–	2.5 (2–3)	–

MoCa, Montreal Cognitive assessment; UPDRS, Unified Parkinson’s Disease Rating Scale; n.a., not applicable.

### MRI acquisition and assessment

MRI was performed with a 3 Tesla scanner (Siemens Prisma, Siemens AG, Erlangen, Germany) using a 20-channel head-neck coil. Image acquisition included the following sequences: 3D T1 MPRAGE (repetition/echo time = 2300/3.43 ms, flip angle = 9°, field of view = 256 mm, slice thickness = 1 mm, voxel size = 1 × 1 × 1 mm), SWI over the midbrain (repetition/echo time 1/echo time 2 = 32/6.08/24.6 ms, flip angle = 15°, FOV 180 mm, slice thickness = 1.5 mm; voxel size = 0.7 × 0.7 × 1.5 mm) and BOLD multiplex echo planar imaging (EPI; repetition/echo time = 901/30 ms, flip angle = 59°, integrated parallel acquisition technique = 2; field of view = 192 mm; # slices = 48; slice thickness (gap) = 3 (0) mm; voxel size = 3 × 3 × 3 mm). The fMRI acquisition protocol was based on previous studies by our group, aiming to maximize signal strength from posterior piriform cortex^15,22^.

Prior to fMRI analysis, T1 images were assessed by a consultant in Neuroradiology (CG, 12 years of experience) for obvious, confounding abnormalities (e.g., infarcts). Nigrosome-1 was assessed on SWI images as previously described^23^. Briefly, nigrosome-1 is a lens-shaped area located caudally in the dorsal part of substantia nigra pars compacta. Nigrosome-1 appears as a hyperintense region in SWI of healthy subjects, but it is hypointense in PD patients due to loss of neuromelanin. Both T1 and SWI were assessed blinded to diagnosis. As mentioned above, one PD patient was excluded from further analysis, due to normal appearance of nigrosome-1 and lacunar infarct in the left caudate nucleus. Two PV patients and one healthy control could not be assessed with SWI, as substantia nigra was only partially visualized. The remaining 13 PV patients and 14 healthy controls showed normal appearance of nigrosome-1.

### Odor stimulation design

Two odors were employed during the task-induced fMRI: peach (FREY&LAU, Henstedt-Ulzburg Germany, product code P0606040) and fish sauce (Thai Fishsauce factory, Sanutsongkharm, 750000 Thailand; article number 08171). Both are relatively selective odorous stimuli producing little or no trigeminal activation^24–27^. In addition, the two odors represent clear odorous concepts which are familiar to most people in Europe. Finally, the pleasant peach odor and the unpleasant fish odor span the most significant domain of the olfactory space. Both odors were used undiluted, in original concentration. Stimulation was administered in blocks of events, with 8 s stimulus length followed by 12 s of resting period. Both odors were randomly embedded in the same session, which included 12 presentations of peach and 6 presentations of fish sauce (total time length 6 min). Given that fish sauce might be perceived as unpleasant by some individuals, we intentionally avoided presenting it as frequently as the generally pleasant aroma of peach. The rationale for juxtaposing these two scents with differing valences was to introduce an element of surprise, enhance alertness, and minimize the likelihood of adaptation, particularly in light of the rapid odor presentation characteristic of our experimental design. The odors were delivered to each participant through a Teflon mask using a computer-controlled olfactometer^28^, embedded in medical air stream (2 l/min airflow), through Teflon-tubing (2 mm inner diameter). Due to larger head size of 4 PD patients and 1 healthy control, it was necessary to use nasal tubes instead of masks during the examination. All other experimental parameters remained the same. To remove residual odors, a constant, inverse airflow was maintained inside the magnet aperture. All participants were instructed to breathe normally through the nose and avoid sniffing.

### CAP methodology

Prior to CAP analysis, all data underwent standard preprocessing steps with the with SPM12 (Wellcome Trust Centre for Neuroimaging, University College London, London, UK). All participants’ images were separately realigned and the translation and rotation correction parameters were individually examined to ensure that no participant had significant head motion larger than one voxel in any direction. Spatial normalization into Montreal Neurological Institute (MNI) space was initially performed on the anatomical T1-weighted image of each participant, and these normalization parameters were then applied to each respective functional image set. The normalized images were smoothed with a 5 mm full width at half maximum Gaussian kernel.

Preprocessed fMRI data were loaded in the CAPs toolbox (TbCAPs, https://github.com/FabienCarruzzo/tbCAPS), as implemented in Matlab, and were further analyzed following the default settings of the developer^18^. Briefly, we employed multi-group analysis and fMRI from each diagnostic group were separately loaded in the toolbox. CAPs were computed on healthy subjects and frames from the PD and PV patients were subsequently assigned to the right CAPs. Also, we employed a multi-seed approach, with right and left posterior piriform cortex being loaded separately on the toolbox, allowing us to compute CAPs even when only one of the posterior piriform cortices was activated. For the posterior piriform cortex, we used previously established masks^29^. We used a threshold T = 0.8 and displacement value M = 0.5 for selecting and scrubbing the z-scored time-series, leaving us with approximately 40% time points with the highest seed activity. Thereafter, after performing K consensus clustering, we decided to delineate two CAPs, as this was the most stable model. For each CAP, the following metrics were derived and used for further statistical comparisons among groups: raw occurrences (total counts of when a CAP is active), average time duration and resilience (probability to remain to a given CAP from time *t* to time *t* + *1*).

### Statistical analysis

Potential differences among groups in terms of demographics (age, MoCA, TDI score) and CAPs metrics (occurrences, time duration, resilience) were assessed with Kruskal–Wallis 1-way ANOVA, with Mann–Whitney U test and Bonferroni correction for pairwise comparisons. Pearson’s correlation was employed to evaluate potentially significant correlations among the subjective hyposmia scale, duration of olfactory dysfunction and each CAP metric. Potential differences in sex were assessed with Fisher’s exact test. As we did not find any significant differences among groups in terms of age, sex or MoCa score (please see the Results section below), we did not use these variables as covariates in subsequent analyses. Significance level was set at p < 0.05 for all tests. All results presented in the text represent the median value with the interquartile range (25% and 75%). Statistical analysis was performed with IBM SPSS Statistics version 29 (Armonk, NY: IBM Corp).

## Results

There were no significant differences in terms of age, sex or MoCa score among the groups. Healthy controls performed significantly better (*p* < 0.01) than both PD and PV patients in all components of the TDI assessment, including the combined TDI score. There were no significant differences between PD and PV patients for any of the TDI components, nor for the combined TDI score. Eight PD patients and three PV patients were classified as functionally anosmic (TDI score ≤ 16.5). The time length of olfactory dysfunction was significantly higher in PD patients than PV patients (*p* < 0.001). However, PV patients reported being significantly more affected by the impaired olfaction (subjective hyposmia scale, *p* < 0.001) compared to PD patients.

**Figure 1:**
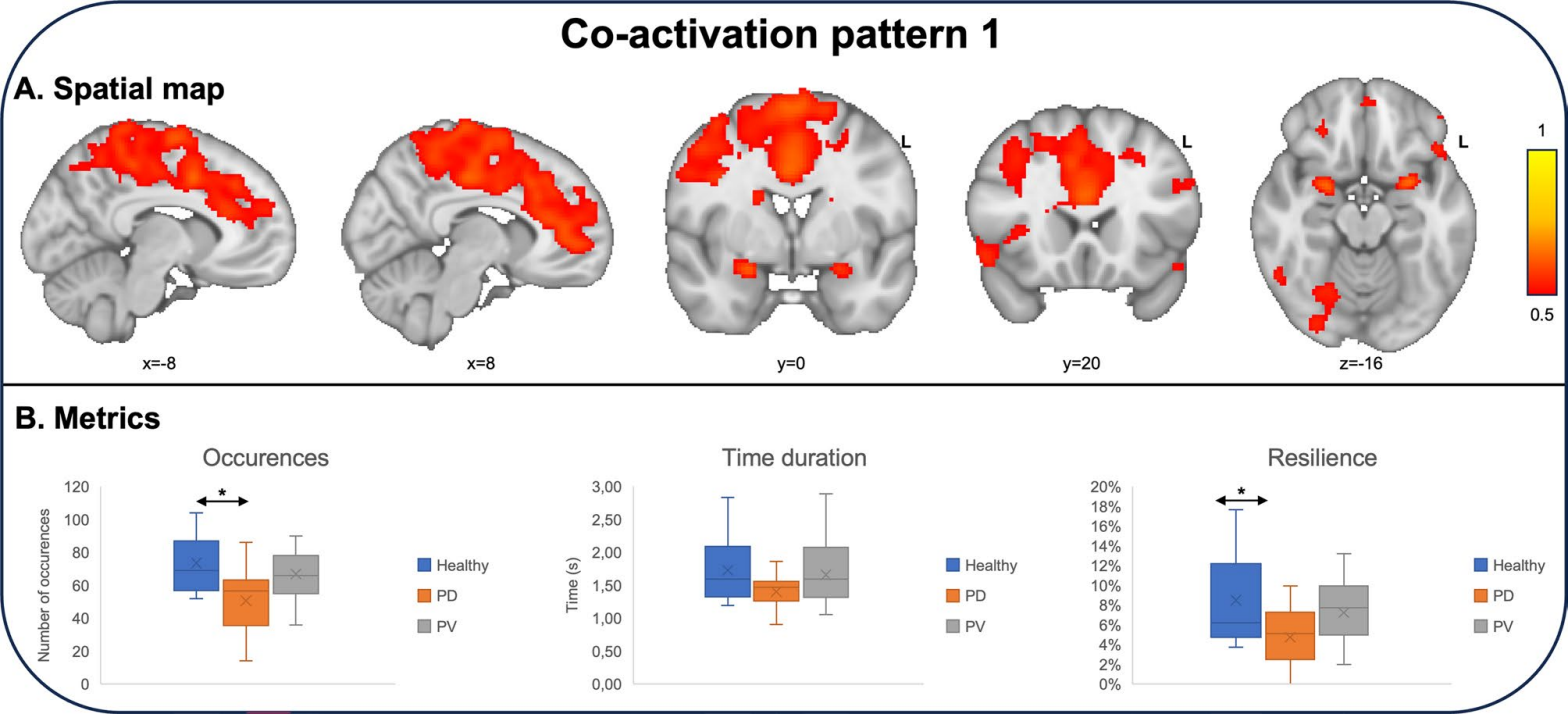
Spatial representation and metrics of the first co-activation pattern (CAP). (**A**) This brain network consisted of posterior piriform cortex (z = − 16, y = 0), anterior cingulate gyrus (x = − 8, x = 8, y = 20), the middle frontal gyrus (y = 0, y = 20) and supplementary motor area (y = 0), the right insula (y = 20) and parts of the orbitofrontal cortex (z = − 16). (**B**) The total number of occurrences and the resilience were significantly lower in PD patients compared to healthy controls (*p* = 0.023 and *p* = 0.016 respectively). There were no significant differences in terms of time duration among groups.

Co-activation pattern analysis showed two distinct networks that interacted with posterior piriform cortex. The first co-activation pattern primarily comprised higher order processing, including the anterior cingulate gyrus, parts of the frontal lobe (specifically the middle frontal gyrus and supplementary motor area), as well as main olfactory projections like the right insula and parts of the orbitofrontal cortex. There was a significantly lower count of occurrences of this pattern in PD patients compared to healthy controls (PD < controls; *p* = 0.023), although no significant differences were observed between healthy controls and PV patients or PV and PD patients. Similarly, there was a significant difference in resilience between healthy controls and PD patients (*p* = 0.016), but no significant differences between healthy controls and PV patients or PV and PD patients. A detailed presentation of the first co-activation pattern, along with its associated metrics, can be found in Fig. 1.

**Figure 2:**
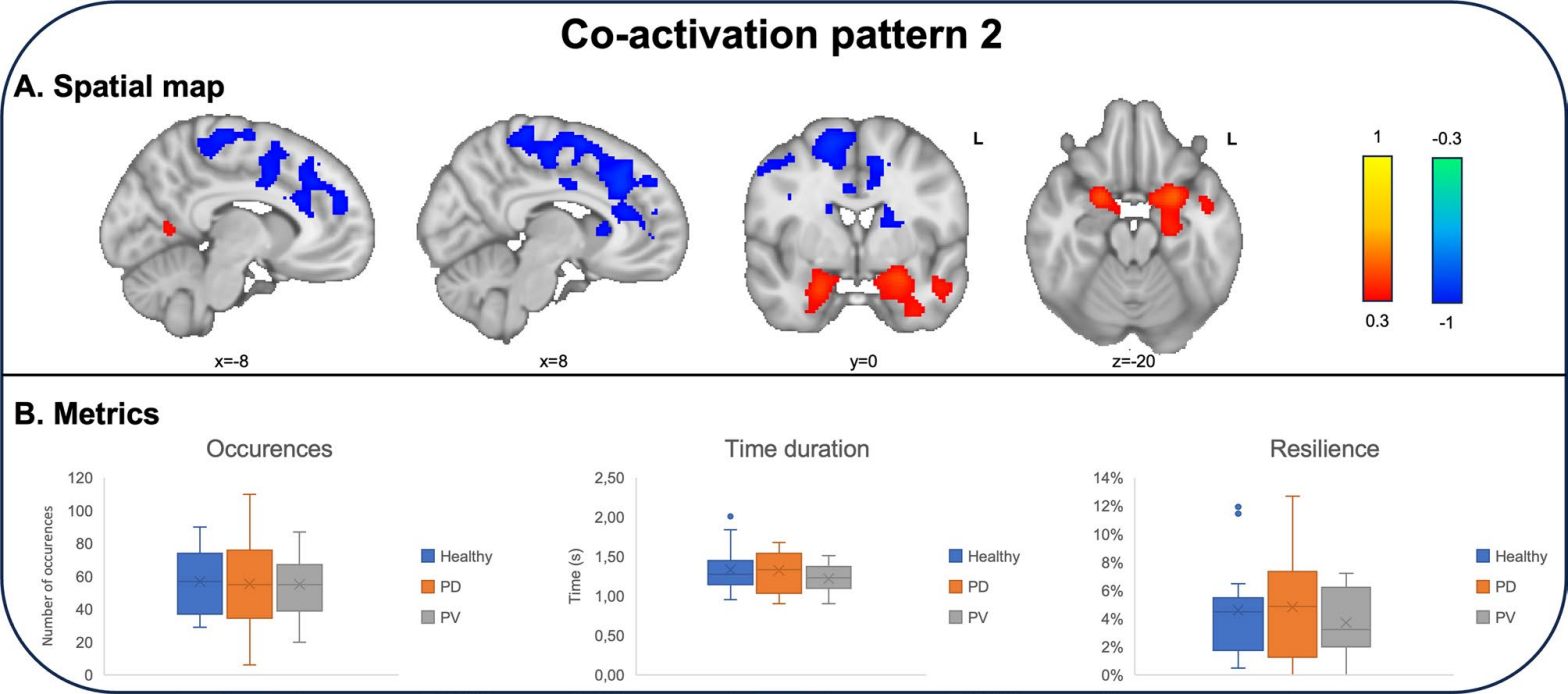
Spatial representation and metrics of the second co-activation pattern (CAP). (**A**) This brain network included the posterior piriform cortex (z = − 20, y = 0), amygdala (z = − 20) and the entorhinal cortex (z = − 20, y = 0). These regions were dissociated from higher order processing in anterior cingulate gyrus (x = − 8, x = 8), middle frontal gyrus (y = 0) and supplementary motor area (y = 0). (**B**) There were no differences in terms of occurrences, time duration or resilience among groups.

The second coactivation pattern encompassed a more extensive area within the olfactory cortex, including the posterior piriform cortex, amygdala and the entorhinal cortex. Interestingly, these regions were dissociated from higher order processing in anterior cingulate gyrus, middle frontal gyrus and supplementary motor area. There were no differences in terms of occurrences, time duration or resilience among healthy controls, PD and PV patients. A detailed presentation of the second co-activation pattern, along with its associated metrics, can be found in Fig. 2.

**Figure 3:**
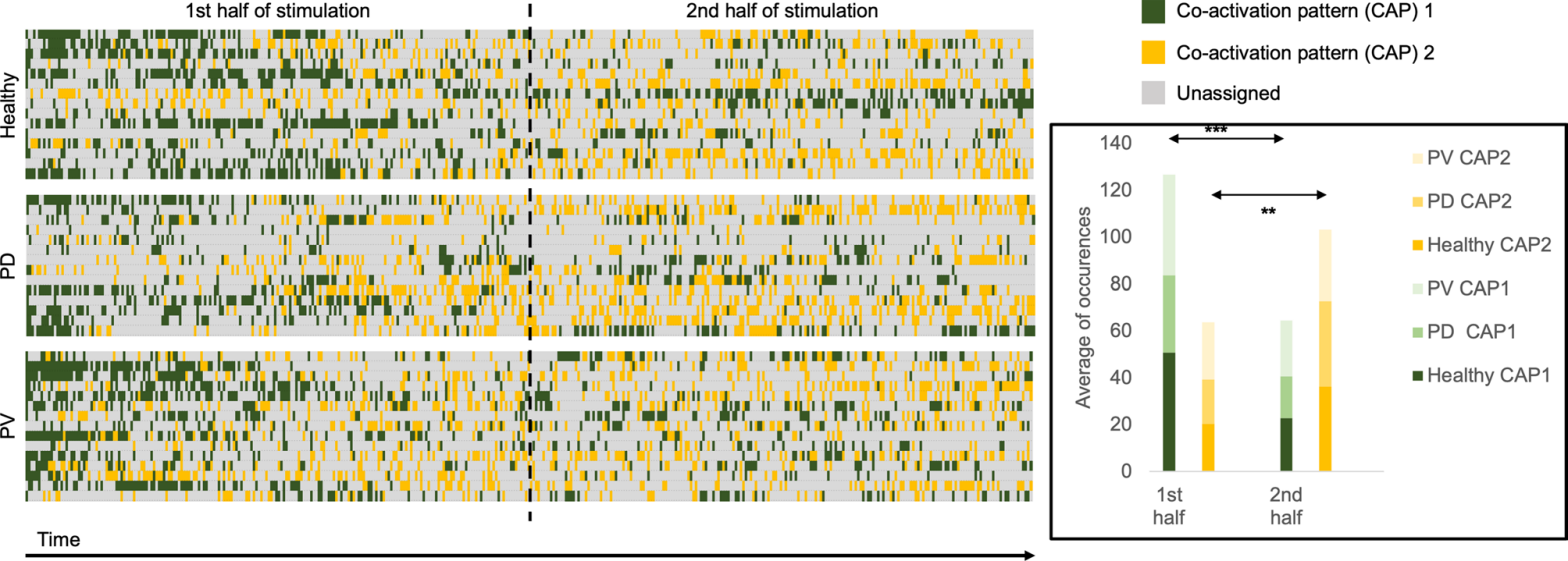
Temporal representation of frame assignment to CAPs. The first CAP occurred significantly more often in the first half of the paradigm across all diagnostic groups (*p* < 0.001), while the second co-activation pattern was significantly occurred more often in the second half of the paradigm (*p* = 0.001).

Temporal analysis of the co-activation patterns revealed distinctive temporal distributions. The first pattern exhibited significantly more occurrences during the first half of the paradigm across all diagnostic groups (*p* < 0.001). Conversely, the second co-activation pattern exhibited significantly more occurrences during the second half of the paradigm across all diagnostic groups (*p* = 0.001). A summary of the temporal analysis results is presented in Fig. 3.

An exploratory correlation analysis between the subjective hyposmia scale and each CAP metric, showed significant positive correlation between subjective hyposmia and raw occurrences of the first coactivation pattern (r = 0.396, *p* = 0.03). We did not find any other significant correlations with either subjective hyposmia or duration of olfactory dysfunction. We further investigated whether peach or fish sauce were predominantly linked to any one of the coactivation patterns. Our analysis found no significant differences in the occurrences of the coactivation patterns in response to either odor.

## Discussion

CAP analysis is a dynamic analytical approach to fMRI data, which can elucidate how a certain brain region interacts with the rest of the brain over the course of an fMRI session. In this study, by using the CAP toolbox, we tried to shed more light on the alterations in connectivity within the posterior piriform cortex under the influence of two distinct conditions that affect olfaction: PD and hyposmia after PV upper respiratory infection. We found a network involving the piriform cortex and higher order processing that was significantly more active in healthy controls than PD patients. Interestingly, PV patients had an intermediate position, with no statistically significant differences observed in comparison to any of the other groups.

Previous olfactory fMRI studies in the context of PD have predominantly relied on traditional analytical approaches. Two previous studies, using the general linear model approach, have reported reduced activity in the amygdalo-hippocampal complex in PD, by employing a general linear model approach^8,30^. Using a similar approach, another study has shown a profound hyperactivation of the piriform and the orbitofrontal cortices in early stages of PD^31^. However, these latter findings have not been replicated. A more recent study showed that the recruitment of a wide-ranging brain network, encompassing both the olfactory cortex and its projections, was significantly less profound in PD patients^7^. The results of our current study align with the majority of previous research, showing decreased activation in PD within a brain network originating from the posterior piriform cortex.

Notably, this network extends beyond the olfactory cortex and its main projections. It also encompasses the anterior cingulate gyrus, a region known for integrating emotion, autonomic, memory and reward-related functions^32^. It also includes the middle frontal gyrus, a brain region that has been associated with literacy, numeracy and attention^33,34^. PD is linked to a wide spectrum of non-motor symptoms, including attention deficits and impairment in the recognition of emotion^35–37^. Our findings suggest a direct association between olfactory processing and the domains of both attention and emotion-related processing. Hence, the lower activation of this network in PD may be attributed not only to olfactory dysfunction but also to deficits in higher order attentional or emotional processing.

While patients with PV-related hyposmia exhibited intermediate metrics of the first coactivation pattern compared to healthy controls and PD patients, there were no significant differences in the recruitment of this network. This could potentially be attributed to the distinct durations of olfactory dysfunction experienced by the two patient groups. PD patients have been living with long-lasting olfactory dysfunction, spanning up to several years prior to the MRI examination, while PV patients have experienced shorter impairments. Therefore, our results suggest the impact of olfactory dysfunction on piriform connectivity in PV patients is manifest, yet less profound, as they did not differ significantly from the other two groups. Interestingly, this finding seems to contrast with the subjective experience of olfactory dysfunction, because the first CAP has been positively correlated with the rated impact of subjective hyposmia. This contradiction may be rooted in the fact that many PD patients are unaware of their olfactory dysfunction, potentially due to the gradual onset of olfactory loss, with adaptation over time^38^. A previous olfactory fMRI study, comparing COVID-19 related olfactory dysfunction with other postinfectious olfactory dysfunction, did not yield any significant differences in orbitofrontal or entorhinal activity^9^.

The second coactivation pattern, occurring predominantly during the latter phase of the stimulation, revealed a dissociation between olfactory cortex and areas for higher order processing, specifically the anterior cingulate gyrus and the middle frontal gyri. This network likely corresponds to olfactory habituation, a phenomenon that has been previously described in olfactory fMRI studies with extended odor stimulation^39,40^. However, our findings are open to different interpretations. The observed decrease in the first coactivation pattern towards the latter half of the experiment, juxtaposed with the increase in the second coactivation pattern, might alternatively be explained by the mere exposure effect. Given the well-established link between repeated exposure and the affective habituation of perception^41^, a clear distinction between these two phenomena in our results is challenging. Given that our cohort primarily comprises patients with impaired olfaction, we intentionally chose a relatively long continuous stimulation, in an effort to ensure activation of the olfactory cortex. Our results suggest that habituation to olfactory stimuli is consistent across all groups, without significant differences in any of the metrics associated with the second coactivation pattern.

One notable limitation of our study is the relatively small cohort size, which however is consistent with other studies in this field. Additionally, our PD group exhibits some degree of heterogeneity, with eight PD patients being classified as functionally anosmic, as opposed to only three patients in the PV group. This heterogeneity may have contributed, at least in part, to the observed results. Moreover, our cohort lacks extensive cognitive, emotional, and attention assessment, which might result in heterogeneity within our patient groups. The absence of comprehensive behavioral data restricts our ability to further look into potential correlations between CAPs and emotion, cognition or attention. Nevertheless, a key strength of our study is the inclusion of nigrosome-1 assessment, ensuring that the majority of healthy controls and PV patients are not in a prodromal phase of PD.

Utilizing state-of-the-art analytical tools for dynamic brain connectivity assessment, our study shows for the first time how the posterior piriform cortex interacts with brain regions that regulate attention and emotions. Importantly, we observe significantly less pronounced recruitment of this brain network in PD patients. Moreover, our findings position patients with PV hyposmia in an intermediate state, with no significant differences from either healthy controls or PD patients. Furthermore, our results highlight the consistent nature of olfactory habituation, a phenomenon seemingly independent of the specific diagnosis. Future studies with similar dynamic analytical approaches are needed in order to further explore the connectivity of olfactory cortex and how this gets influenced by disease.

## Data Availability

The research data can become available on reasonable request.

## References

[1] Brai E, Hummel T, Alberi L (2020). Smell, an underrated early biomarker for brain aging. Front. Neurosci..

[2] Haehner A (2009). Prevalence of smell loss in Parkinson's disease: A multicenter study. Parkinsonism Relat. Disord..

[3] Seiden AM, Duncan HJ (2001). The diagnosis of a conductive olfactory loss. Laryngoscope.

[4] Hummel T (2009). Effects of olfactory training in patients with olfactory loss. Laryngoscope.

[5] Whitcroft KL (2023). Position paper on olfactory dysfunction: 2023. Rhinology.

[6] Sulzer D (2020). COVID-19 and possible links with Parkinson's disease and parkinsonism: From bench to bedside. NPJ Parkinsons Dis..

[7] Georgiopoulos C (2019). A study of neural activity and functional connectivity within the olfactory brain network in Parkinson's disease. Neuroimage Clin..

[8] Westermann B (2008). Functional imaging of the cerebral olfactory system in patients with Parkinson's disease. J. Neurol. Neurosurg. Psychiatry.

[9] Yildirim D, Kandemirli SG, Tekcan Sanli DE, Akinci O, Altundag A (2022). A comparative olfactory MRI, DTI and fMRI study of COVID-19 related anosmia and post viral olfactory dysfunction. Acad. Radiol..

[10] Friston KJ (1994). Statistical parametric maps in functional imaging: A general linear approach. Hum. Brain Mapp..

[11] Wang J, Eslinger PJ, Smith MB, Yang QX (2005). Functional magnetic resonance imaging study of human olfaction and normal aging. J. Gerontol. A Biol. Sci. Med. Sci..

[12] Morrot G, Bonny JM, Lehallier B, Zanca M (2013). fMRI of human olfaction at the individual level: interindividual variability. J. Magn. Reson. Imaging.

[13] Pellegrino R (2016). Olfactory function in patients with hyposmia compared to healthy subjects: An fMRI study. Rhinology.

[14] Vasavada MM (2017). Central olfactory dysfunction in Alzheimer's disease and mild cognitive impairment: A functional MRI study. J. Alzheimers Dis..

[15] Georgiopoulos C (2018). Olfactory fMRI: Implications of stimulation length and repetition time. Chem. Senses.

[16] Beckmann CF, Smith SM (2004). Probabilistic independent component analysis for functional magnetic resonance imaging. IEEE Trans. Med. Imaging.

[17] Hutchison RM (2013). Dynamic functional connectivity: Promise, issues, and interpretations. Neuroimage.

[18] Bolton TAW (2020). TbCAPs: A toolbox for co-activation pattern analysis. Neuroimage.

[19] Rey G (2021). Dynamics of amygdala connectivity in bipolar disorders: a longitudinal study across mood states. Neuropsychopharmacology.

[20] Paakki JJ (2021). Co-activation pattern alterations in autism spectrum disorder-A volume-wise hierarchical clustering fMRI study. Brain Behav..

[21] Gottfried JA (2010). Central mechanisms of odour object perception. Nat. Rev. Neurosci..

[22] Eek T, Lundin F, Larsson M, Hamilton P, Georgiopoulos C (2023). Neural suppression in odor recognition memory. Chem. Senses.

[23] Haller S, Davidsson A, Tisell A, Ochoa-Figueroa M, Georgiopoulos C (2021). MRI of nigrosome-1: A potential triage tool for patients with suspected Parkinsonism. J. Neuroimaging.

[24] Zang Y, Chen B, Hummel T (2020). Assessment of odor perception related to stimulation modes in a mock MRI scanner. J. Neurosci. Methods.

[25] Zang Y (2021). Brain response to odors presented inside the nose, directly in front of the nose or with ambient air. Eur. Arch. Otorhinolaryngol..

[26] Han P, Chen H, Hummel T (2020). Brain responses to food odors associated with BMI change at 2-year follow-up. Front. Hum. Neurosci..

[27] Sorokowska A, Hummel T, Oleszkiewicz A (2020). No olfactory compensation in food-related hazard detection among blind and deaf adults: A psychophysical approach. Neuroscience.

[28] Sommer JU (2012). A mobile olfactometer for fMRI-studies. J. Neurosci. Methods.

[29] Seubert J, Freiherr J, Djordjevic J, Lundstrom JN (2013). Statistical localization of human olfactory cortex. Neuroimage.

[30] Hummel T (2010). Olfactory FMRI in patients with Parkinson's disease. Front. Integr. Neurosci..

[31] Moessnang C (2011). Altered activation patterns within the olfactory network in Parkinson's disease. Cereb. Cortex.

[32] Stevens FL, Hurley RA, Taber KH (2011). Anterior cingulate cortex: unique role in cognition and emotion. J. Neuropsychiatry Clin. Neurosci..

[33] Japee S, Holiday K, Satyshur MD, Mukai I, Ungerleider LG (2015). A role of right middle frontal gyrus in reorienting of attention: a case study. Front. Syst. Neurosci..

[34] Koyama MS, O'Connor D, Shehzad Z, Milham MP (2017). Differential contributions of the middle frontal gyrus functional connectivity to literacy and numeracy. Sci. Rep..

[35] Gray HM, Tickle-Degnen L (2010). A meta-analysis of performance on emotion recognition tasks in Parkinson's disease. Neuropsychology.

[36] Krauzlis RJ, Bollimunta A, Arcizet F, Wang L (2014). Attention as an effect not a cause. Trends Cogn. Sci..

[37] Bora E, Walterfang M, Velakoulis D (2015). Theory of mind in Parkinson's disease: A meta-analysis. Behav. Brain Res..

[38] Haehner A, Hummel T, Reichmann H (2011). Olfactory loss in Parkinson's disease. Parkinsons Dis..

[39] Sobel N (2000). Time course of odorant-induced activation in the human primary olfactory cortex. J. Neurophysiol..

[40] Poellinger A (2001). Activation and habituation in olfaction: An fMRI study. Neuroimage.

[41] Ferdenzi C, Poncelet J, Rouby C, Bensafi M (2014). Repeated exposure to odors induces affective habituation of perception and sniffing. Front. Behav. Neurosci..

